# Cannabinoids and their derivatives in struggle against melanoma

**DOI:** 10.1007/s43440-021-00308-1

**Published:** 2021-07-15

**Authors:** Paweł Marzęda, Małgorzata Drozd, Paula Wróblewska-Łuczka, Jarogniew J. Łuszczki

**Affiliations:** Department of Pathophysiology, Medical University of Lublin, Jaczewskiego 8b, 20-090 Lublin, Poland

**Keywords:** Cannabinoids, Melanoma, Antitumor effect, CB1 receptor, CB2 receptor

## Abstract

**Abstract:**

Melanoma is one of the most aggressive malignances in human. Recently developed therapies improved overall survival rate, however, the treatment of melanoma still remains a challenging issue. This review attempts to summarize recent advances in studies on cannabinoids used in the setting of melanoma treatment. Searches were carried out in PubMed, Google Scholar, Scopus, Research Gate. Conclusions after analysis of available data suggest that cannabinoids limit number of metastasis, and reduce growth of melanoma. The findings indicate that cannabinoids induce apoptosis, necrosis, autophagy, cell cycle arrest and exert significant interactions with tumor microenvironment. Cannabinoids should be rather considered as a part of multi-targeted anti-tumor therapy instead of being standalone agent. Moreover, cannabinoids are likely to improve quality of life in patients with cancer, due to different supportive effects, like analgesia and/or anti-emetic effects. In this review, it was pointed out that cannabinoids may be potentially useful in the melanoma therapy. Nevertheless, due to limited amount of data, great variety of cannabinoids available and lack of clinical trials, further studies are required to determine an exact role of cannabinoids in the treatment of melanoma.

**Graphic abstract:**

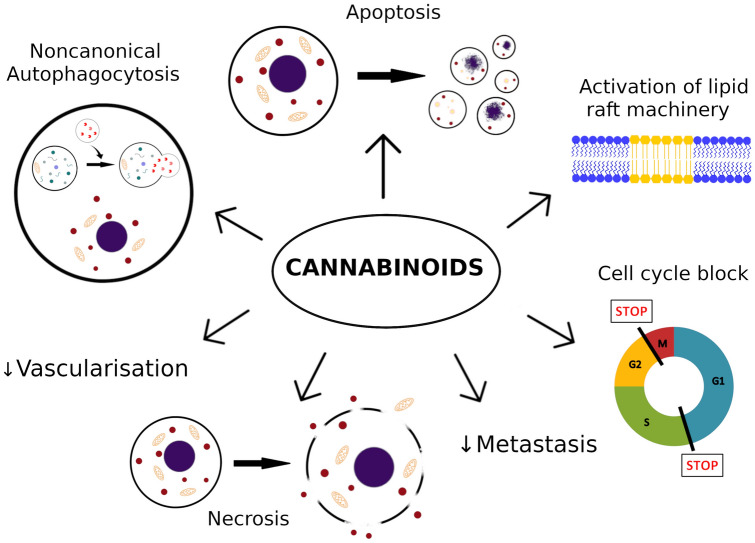

## Introduction

The endocannabinoid system is dysregulated in numerous pathological conditions, including malignancies. These alterations might include function and/or expression of cannabinoid receptors and enzymes, or affect concentration of various cannabinoid receptor ligands [1]. Recently, cannabinoids have received increasing amount of interest in the setting of treatment of various cancers. However, majority of data comes from in vitro and animal studies, therefore, most of potential uses of cannabinoids still require validation in patients.

The use of cannabinoids is a widely debated crucial issue. Currently, the medical cannabis is legalized for medical use in 19 European Union countries, Canada and 36 states in US [2, 3]. In EU, law permits use of cannabis based drugs for various conditions and symptoms such as cancer treatment, chronic pain, nausea, anorexia, muscle spasticity, AIDS, multiple sclerosis, and seizures [2]. Cannabis, however, contains not only cannabinoids, but also terpenes and flavonoids. These different compounds of cannabis were also reported to exert the anti-tumorigenic actions [1]. The best studied groups of substances isolated from cannabis are cannabinoids. Their therapeutic potential has been observed in several malignancies, including breast, prostate, lung, skin, pancreatic and bone cancers, as well as lymphoma and glioma [4]. In last few years, efforts have been made to determine the role of cannabinoids in the setting of melanoma treatment.

### Melanoma: epidemiology, prognosis, risk factors

Melanoma represents 1.6–5.5% of all cancers [5, 6]. Unlike most of the neoplasms, it often affects young people. It accounts for majority of deaths caused by skin cancers in total. Globally, in 2018, there were approximately 324,635 new cases of melanoma and 57,043 deaths caused by it in 185 countries [5, 6]. In the last few years, there was significant mortality decline in melanoma, due to new therapies for metastatic disease [6]. Nevertheless, melanoma is one of the most aggressive malignancies. It harbors one of the highest mutation frequencies among human cancers [7]. The leading driver of mutagenesis in melanoma is ultraviolet light. Tremendous mutation burden correlates with response to different therapies [8]. Once metastases occur, the prognosis is considered to be poor. On the genetic level, crucial determinant of anti-tumor immune response to melanoma is tumor heterogeneity. Two major origins of aggressiveness of melanoma are: degree of genetic diversity of the tumor and number of distinct clones composing it. These factors impede treatment of metastatic melanoma and are linked to response to the immune therapy and patients’ survival [9].

The main risk factor for developing melanoma is excessive exposure to ultraviolet radiation. The highest risk occurs in individuals with a phenotype of blond or red-hair, light-colored eyes, freckles and pale skin. Moreover, presence of multiple dysplastic or benign melanocytic neavi, immunosuppression, positive family history and skin sunburn, especially during childhood and adolescence also contribute to the development of melanoma [10, 11].

### Treatment options of melanoma

The most important way to reduce mortality in melanoma is its early detection. Therapy of localized melanoma is well established and primarily based on wide local surgical excision with proper margins [12]. The 5-year survival rate at this stage reaches 99% [13]. Management of metastatic melanoma has been dramatically reshaped over last decade. From usage of conventional chemotherapy like dacarbazine, it evolved into immunotherapy and molecularly targeted therapies [14]. Main and most effective agents are immune checkpoint inhibitors, including anti-programmed death-1 (anti-PD1) and anti-cytotoxic T lymphocyte antigen-4 (anti-CTLA4) antibodies. Implementation of these agents resulted in increase of 5-year survival rate from 15 to 20% up to 52%, but at cost of high toxicity [15, 16]. Moreover, in patients with the *BRAF* V600E mutation, the antibodies are likely to have high response rates from use of molecularly targeted, the therapies serine-threonine kinases inhibitors (inhibitors of BRAF, MEK, KIT) [17]. However, the response to these agents is generally less durable and a 5-year survival rate reached maximum 28% in patients with metastatic melanoma treated with the most favorable BRAF and MEK inhibitors combination [18]. This therapy, however, seems important for younger patients (aged < 40 years), who are more likely to have *BRAF* V600 mutation, which allows predicting sensitivity to inhibitors of BRAF and MEK, as do *KIT* exon 11 mutations to KIT inhibitors [8]. Overall, the combined therapy with BRAF and MEK inhibitors is superior within the first 6 months, becoming after that point inferior to PD-1 blockers alone or in combination with CTLA-4 blockers [19]. Efficiency of current therapies is often limited due to their toxicity, undesirable side effects, frequent metastases and quickly growing resistance mechanisms [20, 21]. Studies on new molecular targets and immunotherapeutic options are still ongoing (Table 1). Along that appeared novel ideas, like an application of oncolytic virus talimogene laherparepvec (T-VEC) [22].

**Table 1:** Overview of cannabinoids’ actions on melanoma

Compound	Cell line (type of study)	Effect	References
CBD	B16F10 (C57BL/6 mice, s.c. injection of 1 × 10^5^ cells)	↓ Tumor growth ↑ Survival time ↑ Quality of life	[68]
THC	B16, HCmel12 (in vitro)	No effect	[62]
THC	B16, A375, MelJuso (in vitro)	↓ Cell viability No effect on normal melanocytes	[64]
THC	B16 (in vivo, Hgf-Cdk4^R24C^, WT and Cnr1/2^−/−^ mice, i.c. injection of 1 × 10^5^ cells)	No effect	[62]
THC	HCmel12 (in vivo, Hgf-Cdk4^R24C^, WT and Cnr1/2^− /−^ mice, i.c. injection of 1 × 10^5^ cells)	↓ Tumor growth ↓ Inflammatory immune cells infiltration tumor angiogenesis) No effect on Cnr1/2^−/−^ mice	[62]
THC/CBD + THC	A375, SK-MEL-28, CHL-1 (in vitro)	↓ Cell viability ↑ Apoptosis (requires TRIB3) Noncanonical autophagy (Atg7-dependent)	[65]
THC/CBD + THC	CHL-1 (in vivo*,* Athymic Nude Mice, s.c. injection of 7.5 × 10^6^ cells)	↓ Tumor size ↑ Noncanonical autophagy	[65]
AEA	A375 (in vitro)	↓ Cell viability ↑ Cytotoxicity ↑ Caspase-dependent apoptosis Potentiated by FAAH inhibition Mitigated by COX-2 and LOX inhibition Possible role of lipid raft and GPR55	[27]
AEA	HT168-M1 (in vitro)	↓ Migration	[57]
AEA	HT168-M1 (in vivo, SCID mice, inoculation into the spleen of 5 × 10^4^ cells/animal)	No effect	[57]
AEA, ACEA, Met-F-AEA	HT-168-M1, WM35, WM983B (in vitro)	↑ Apoptosis Cell necrosis in higher concentrations G2/M block	[57]
PEA	B16 (in vitro)	↑ Cytotoxicity ↑ Apoptosis ↓ Cell viability	[70]
PEA + URB597	B16 (in vitro)	↑ Apoptosis ↑ Necrosis ↓ Cell viability	[70]
PEA + URB597	B16 (in vivo, C57BL/6 mice, s.c. injection of 10^6^ cells)	↓ Tumor growth ↑ Necrosis No antiangiogenic effects	[70]
ACEA	OCM-1A, COLO38 (in vitro)	No effect	[26]
ACEA	HT168-M1 (in vivo, SCID mice, inoculation into the spleen of 5 × 10^4^ cells/animal)	↓ Metastasis ↓ Migration and colonization No effect on tumor growth	[57]
AM251	HT-168-M1, WM983B (in vitro)	↑ Apoptosis G2/M block	[57]
AM251	A375 (in vitro)	No effect	[27]
WIN 55,212–2	B16, A375, MelJuso (in vitro)	↓ Cell viability No effect on normal melanocytes	[64]
WIN 55,212–2	OCM-1A, COLO38 (in vitro)	↑ Apoptosis ↓ Cell viability Action via lipid raft machinery (involves cleavage of caspases 9 and 7, ERK phosphorylation)	[26]
WIN 55,212–2	B16 (in vivo, C57BL/6 mice, s.c. flank injection of 1 × 10^5^ cells)	↓ Tumor growth ↓ Metastasis ↓ Cell proliferation ↓ Tumor vascularization ↑ Apoptosis Inhibition of cell cycle at G1-S transition	[64]
JWH-133	OCM-1A, COLO38 (in vitro)	No effect	[26]
JWH-133	A2058 (in vitro)	↓ Migration rate and adhesion Involvement of Gi/Goα subunits Downregulation of ICAM, VCAM and MMP	[63]
JWH-133	B16 (in vivo, C57BL/6 mice, s.c. injection of 1 × 10^5^ cells)	↓ Tumor size ↓ Tumor vascularization ↑ Apoptosis Inhibition of cell cycle at G1-S transition	[64]
JWH-133 + ACEA	OCM-1A, COLO38 (in vitro)	No effect	[26]

*ACEA* arachidonyl-2-chloroethylamide; *ACPA* arachidonylcyclopropylamide; *AEA* anandamide; *anti-CTLA4* anti-cytotoxic T lymphocyte antigen-4; anti-*PD1* anti-programmed death-1; *AMP* adenosine monophosphate; *CB1* cannabinoid receptor type 1; *CB2* cannabinoid receptor type 2; *CBD* cannabidiol; *Cnr1/2*^*−/−*^ CB1/CB2 receptor-deficient; *COX* cyclooxygenase; *ERK* extracellular signal-regulated kinase; *FAAH* fatty acid amide hydrolase; *GIRK* G protein-coupled inwardly-rectifying potassium channel; *GPCR* G protein-coupled receptors; *i.c.* intracutaneously; *ICAM* intercellular adhesion molecule; *JNK* c-Jun N-terminal kinase; *LOX* lipooxygenase; *MAPK* mitogen-activated protein kinase; *Met-F-AEA* 2-methyl-arachidonyl-2′-fluoro-ethylamide; *MMP* metalloproteinases; *NAAA* *N*-acylethanolamine-hydrolyzing acid amidase; *PEA* *N*-palmitoylethanolamine, *PPAR* peroxisome proliferator activated receptor, *s.c.* subcutaneously, *SCID* severe combined immunodeficiency, *THC* tetrahydrocannabinol, *TRP* transient receptor potential, *VCAM* vascular cell adhesion molecule, *VR-1* vanilloid receptor 1, *WT* wild type

### Cannabinoids: mechanisms of action and pathways

Cannabinoids exert their actions mainly by binding to G-protein-coupled CB1 and CB2 receptors and other receptors, such as peroxisome proliferator activated receptors PPARs), transient receptor potential (TRP) channels and other G-protein-coupled orphan receptors, like GPR18, GPR55 or GPR119 and serotonin 1A receptor (5-HT1A) [23–25]. Moreover, a role of lipid raft cannot be excluded [26, 27]. Hundreds of phyto- and synthetic cannabinoids demonstrate diverse pharmacological effects on the particular cell types by acting as agonists or antagonists/inverse agonists of CB1 and CB2 receptors, however, only a few have found their place in clinical use [28].

The cannabinoid CB1 receptors are expressed in neurons of the central nervous system, peripheral nerve terminals and non-neuronal tissues, such as adipose tissue, lungs, liver, spleen, uterus and testis among others [29, 30]. In general, the activation of CB1 receptors is responsible for psychiatric effects, such as alterations in movement, sensory learning, analgesia, anxiety, appetitive behaviors and abuse of cannabinoids. Additionally, CB1 agonists reveal a variety of effects—anti-depressant, anti-nociceptive, anti-convulsant, anti-depressant, anxiolytic, anti-emetic, orexigenic, anti-proliferative and anti-migration. The CB1-selective antagonists/inverse agonists have anti-diabetes effect and may be helpful in reducing body mass and treating drug dependency [28].

The cannabinoid CB2 receptors are mainly expressed in the immune system cells [31]. The activation of CB2 receptors is responsible for cannabinoids’ anti-inflammatory, immunomodulatory, anti-cancer, anti-spasmatic, anti-nociceptive, neuroprotective, osteoprotective and anti-obesity effects [28, 32, 33]. Against common belief, the CB2 receptors are expressed in the central nervous system, however, their role is not yet fully understood. Their expression in the central nervous system and other tissues seems to be upregulated in some of pathological conditions [34, 35]. The CB2-selective agonists have been paid a lot of attention lately, because of their therapeutic potential in treating pain, inflammation, neuroinflammatory or neurodegenerative diseases and cancer, while avoiding the psychotropic effects related to the CB1 receptor activation [28, 36]. Most of them are investigated in preclinical studies with success, but only few of them reached the stage of clinical trials [28, 37].

Both, CB1 and CB2 receptors are members of the G protein-coupled receptors (GPCRs) and primarily couple to pertussis toxin-sensitive G proteins of the Gi and Go classes [38]. They suppress adenylyl cyclase, thereby formation of cyclic AMP, induce activation of mitogen-activated protein kinase (MAPK) pathways [38, 39]. Moreover, stimulation of CB1 receptor activates extracellular signal-regulated kinase 1 or 2 (ERK1/2), p38 MAPKs and c-Jun N-terminal kinases (JNKs) [30, 39]. Besides MAPKS, CB1 receptor also activates the PI3K/Akt pathway [30, 40, 41]. Due to limited number of studies on CB2 receptor activation of MAPK and other pathways, data in that area is limited. The CB1 receptor was reported to activate other G proteins in particular cell types in the ligand-dependent manner [39, 42]. Another difference between CB1 and CB2 receptors is that that the former receptors act through modulation of activity of ion channels. They activate G-protein-coupled inwardly rectifying potassium channel (GIRKs), inhibit N-type calcium channels and have various effect on L-type calcium channels [42–44].

The precise description of cannabinoid receptor signaling is complex and exceeds the scope of this article. The more detailed information on signal transduction pathways have been described elsewhere [39, 42, 43, 45].

### Phytocannabinoids and synthetic analogs

Cannabinoids are the major compounds of the *Cannabis sativa* L. plant, which psychotropic and therapeutic properties have been used by mankind since thousands of years. *Cannabis* plant contains more than 120 different active constituents, named phytocannabinoids. The pharmacological effect of whole plant extract intake is the sum of compounds’ effects and is inconstant due to the varying proportions of phytocannabinoids in different environment. Phytocannabinoids used in research on melanoma are: Δ9-tetrahydrocannabinol (THC) and cannabidiol (CBD) [46].

The biologically active synthetic analogues of cannabinoids used in research on melanoma belong to the group of Δ^9^-THC-like analogs or aminoalkylindole compounds. JWH-133 (as a synthetic derivative of THC) is a potent analog with high affinity to CB2 receptors. WIN55212-2 (as aminoalkylindole derivative) is agonist of both CB1 and CB2 receptors [38].

Anandamide (AEA) is an endogenous cannabinoid, partial agonist of CB1 and CB2 receptors. Its synthetic analogues, more selective towards CB1 receptor, are: arachidonyl-2′-chloroethylamide (ACEA) and arachidonylcyclopropylamide (ACPA). The other endogenous cannabinoid, fatty acid amine, is palmitoylethanolamide (PEA) [38].

AM251, one of the arylpyrazoles, is an inverse agonist of CB1 receptors, usually used to block CB1 receptor-mediated effects [38]. The effects of these compounds regarding to melanoma are reviewed below.

### Cannabinoids-related adverse effects

The CB1 receptor due its high expression in central nervous system is the main receptor responsible for psychotropic effects caused by cannabinoids. Because CB1 receptors are widespread in mammalian tissues, therefore, their activation leads to numerous adverse effects through the body and limits its therapeutic application. Crucial side effects of CB1 receptor ligands include neurological disorders, cardiovascular dysfunction, digestion failure and potential for addiction [28, 30]. On the other hand, CB1 receptor antagonists/inverse agonists cause gastrointestinal disorders and psychiatric disturbances, such as depression, anxiety, and suicidal ideation [28, 47].

The CB2 receptor agonists were reported to be well tolerated [48, 49]. Possible side effect of CB2 receptors activation, due to their predominance in immune cells is immune dysfunction and immunosuppression [50]. Lately possible adverse effects of CB2 receptor agonists on reproductive system have been reported, i.e. decreased sperm count, impairment of placental development and reduced offspring growth [51]. Nonetheless, adverse effects caused by the particular substances are more complex, due to their various effects on different receptors and off targets actions. For instance, the application of cannabidiol (CBD) causes diarrhea, decreased appetite and somnolence, pyrexia and vomiting [52]. WIN55,212-2 application may lead to anxiety, recognition memory impairment and brain network functional connectivity impairment [53, 54].

In last years the issue of determining cannabinoids’ therapeutic potential in different neoplasms emerged, which seems important to be evaluated. The aim of this review is to point out the mechanisms of action of cannabinoids and determine if cannabinoids have a potential to be included into the treatment of melanoma.

## Materials and methods

The literature review was undertaken by searching following terms in combination (“Cannabinoids” OR “Cannabis sativa” OR “CBD” OR “Cannabidiol” OR “THC” OR “Tetrahydrocannabinol” OR “AEA” OR “anandamide” OR “ACEA” OR “arachidonylcyclopropylamide” OR “PEA” OR “palmitoylethanolamide” OR “AM251” OR “JWH-133” OR “URB597” OR “WIN 55,212-2” OR “2-AG” OR “arachidonoyl glycerol”) AND (“melanoma” OR “melanocarcinoma” OR “melanosarcoma” OR “melano-epithelioma” OR “A375”or “SK-MEL” OR “FM55” OR “B16” OR “HCmel12”) on electronic databases, PubMed, Scopus, and additionally Research Gate and Google Scholar. The results were limited to December 2020. Relevant studies’ reference lists were also hand-searched. The inclusion criteria were research studies on the action of cannabinoids on melanoma only, both in vivo and in vitro. The exclusion criteria were: reviews, commentaries and non-English articles. All the abstracts and full texts were reviewed independently by two authors (P.M. and M.D.) focusing on criteria of inclusion and exclusion. Totally, 414 records were detected using electronic databases. After screening, 376 articles were excluded as irrelevant and 38 articles were assessed for eligibility. The relevant studies were selected by second full-text screening. After removing duplicates and assessment, only nine experimental studies were included in this review.

The main limitations in this review are those related with selection of previously published original studies (methods of search outlined above and appropriateness of these research papers with the inclusion/exclusion criteria).

## Results

Numerous mechanisms of action of endocannabinoid system have been described in different carcinomas [55]. The debate on the role of elevated levels of endocannabinoids and increased cannabinoid CB receptors’ expression in neoplasms is still ongoing [56]. Nevertheless, the knowledge of how do cannabinoids work in melanoma is still limited, yet it is progressing. Multiple studies report that melanoma expresses CB1 and CB2 receptors, and other receptors like GPR family or TRPV1 [27].

*Role of the CB1 receptors* The CB1 receptors in melanoma cells were localized in the membrane, cytoplasm and cytoskeleton. Genes of this receptor (CB1) were proved to be similar in different, unrelated melanoma cell lines [57]. They pay considerable contribution to the anti-tumor effects of cannabinoids. Activation of CB1 receptors causes significant induction of apoptosis, arrests cells in the G2/M phase of the cell cycle and, in higher concentrations of CB1 receptor agonists, might even lead to cell necrosis. They also contribute to the anti-migratory effect of cannabinoids. Moreover, the combinations of various CB1 agonists and inverse agonists lead to additive anti-proliferative effects [57]. However, in one study, it was suggested that the CB1 receptors may contribute to the development of melanoma, particularly in A375 and 501Mel cell lines, by promoting cell growth, migration, clonogenicity, cell cycle progression and activation of ERK and Akt signaling pathways [58]. It is supported by the observation that silencing of the CB1 receptor or delivering CB1 antagonist like SR141716 induces cell cycle arrest in the G1/S-phase in other tumors, while it does not lead to apoptosis or necrosis [59, 60]. Gathered data suggest that CB1 agonists and antagonists may work by off-target mechanisms, making the interpretation complex and ambiguous [26, 58].

*Role of CB2 receptors* The CB2 receptors are overexpressed in the melanoma cells, but not expressed in normal surrounding tissues [61]. It is suggested that CB2 receptors might be associated with melanoma development and activators of CB2 receptors may be used in the treatment of this skin cancer [61]. In contrast, one study presented that absence of the CB1 and CB2 receptors did not affect development of chemically induced skin tumors [62]. Besides, the activation of CB2 receptors can also improve blood–brain barrier function via endothelial cells, which may reduce number of melanoma brain metastases. This effect is achieved by reduction of adhesion and trans-endothelial migration of melanoma cells. The best effect of over 40% reduction was observed when both, brain endothelial cells and melanoma cells were pre-treated with the CB2 agonist—versus 25–30% reduction without pretreatment [63].

### Cannabinoid derivatives in the treatment of melanoma

#### Δ9-Tetrahydrocannabinol (THC)

 THC mimics endocannabinoids and binds to both CB1 and CB2 receptors, as partial agonist. Therefore, it presents the mixed agonist–antagonist effect which is likely to be dependent on the expression of CB receptors, presence of agonists or endocannabinoids and the cell types [46]. THC causes loss of cell viability in a dose-dependent manner by activation of apoptosis and autophagy in melanoma cells both, in vitro and in vivo (Table 1). THC causes death of malignant melanoma cells in concentrations, which are safe for normal melanocytes [64, 65]. Viability of most of non-transformed cells seems to be not as sensitive on cannabinoids as cancerous cells, however, highly proliferative cells may undergo apoptosis and cell death [64, 66]. In another study, the THC treatment in the mouse model significantly reduced HCmel12 cell line growth but, THC did not affect B16 cell line and mice lacking the CB1 and CB2 receptors till the end point of the study [62]. Moreover, there was no therapeutic effect in in vitro models, which stands in contrast to other conducted studies [64, 65]. Lack of the action might have occurred due to very low expression levels of CB1/CB2 receptors in this model, suggesting the important role of CB1/CB2 receptors in inhibiting melanoma.

#### WIN 55,212-2

 Activation of CB receptors by the mixed CB1 and CB2 agonist—WIN 55,212-2 can significantly block the formation of new blood vessels, decrease proliferation, tumor growth and induce apoptosis of melanoma cells in the mouse exografted tumor model (Table 1). The main proposed mechanism of action in this model was rapid inhibition of the pro-survival Akt pathway via tumor suppressor retinoblastoma protein, resulting in cell cycle arrest at G1-S transition. These effects, together with reduced metastasis are independent on immune status of animals and are similar to THC, selective for melanoma cells versus normal melanocytes [64]. Another potential mechanism of the anticancer action of WIN 55,212-2 involves membranes lipids. In one of the studies, WIN 55,212-2 caused melanoma cell death independent on CB1, CB2 and VR-1 receptors, via lipid raft machinery [26]. The possible role of lipid rafts was also stated during anandamide (AEA) tests [27].

#### Cannabidiol

 CBD has very low affinity to both CB1 and CB2 receptors and acts as antagonist/inverse agonist of the CB1, a partial agonist of the CB2 and negative allosteric modulator of the CB1 receptors, and at sub-micromolar concentrations, as an antagonist of both CB1 and CB2 receptors [46]. Multiple receptors affected by CBD and various mechanisms take part in modulation of oncogenic signaling and redox homeostasis [67] (Table 1). Recent study presents beneficial therapeutic effect of CBD in a murine model. The administration of CBD in mice with injected subcutaneously melanoma caused significant extension of survival time and decrease in tumor growth compared to the control animals without treatment [68]. The combination of THC with CBD causes stronger inhibition of cell viability in both in vitro and in vivo studies [65].

#### Anandamide (AEA), its analogues and inhibitors

 AEA is the CB1/CB2 receptor agonist, TRPV1 agonist, putative GPR55 agonist, induced in the A375 melanoma cell line a concentration-dependent cytotoxicity and decrease in cells viability (Table 1). Potential factors involved in action of AEA were by-products of its metabolism derived by cyclooxygenase-2 (COX-2) and lipooxygenase (LOX), and possibly contribution of lipid raft modulation, GPR55 and CB1 receptors. Effect of AEA was mitigated by AM251 (a CB1 receptor antagonist/inverse agonist), which did not affect cell viability by itself. Due to the lack of CB2 receptors in used cell line and no effect of a selective CB2 receptor agonist (JWH133) on cell viability, the effect of AEA was attributed mainly to AEA by-products and CB1 receptors [27]. It is worth to note that in this particular cell line the lack of CB2 receptors may be ascribed to clonal difference, because in other A375 melanoma cell lines, the CB2 receptor was expressed [64], or even overexpressed [61].

In another study, AEA and its more selective towards CB1 receptor analogues, the arachidonyl-2′-chloroethylamide (ACEA) and 2-methyl-2′-F-anandamide (Met-F-AEA) exerted the anti-proliferative effect on Ht168-M1 and WM983B melanoma cells. This action was assigned to induction of apoptosis, G2/M arrest and cell necrosis [57]. In contrast to previous study, AM251 (an inverse agonist at the CB1 receptor) was observed to have even stronger pro-apoptotic effect and to cause G2/M phase cell cycle arrest [57, 69]. On the other hand, one of these studies confirmed that AM251 potentiates the effect of CB1 agonists [57]. It was found that B16 melanoma cells produce enzymes that degrade endocannabinoids and the blockade of hydrolysis by various inhibitors, including URB597 (a relatively selective inhibitor of the enzyme fatty acid amide hydrolase (FAAH)), which leads to increase of endocannabinoids levels and even greater increase of cytotoxicity of AEA and palmitoylethanolamide (PEA). The most potent combination was PEA and URB597, when using together causes higher apoptosis and necrosis rates in vitro than each of the tested agents alone*.* It was confirmed in in vivo model of B16 melanoma in C57BL/6 mice, where PEA-URB597 combination reduced growth of the tumor and reduced its size [70].

#### JWH-133

 The selective CB2 agonist JWH-133 was reported in one study to exert no effect on melanoma in vitro [26], but in another, it inhibited tumor growth and decreased cell proliferation in vivo [64] (Table 1).

The above-mentioned studies suggest a significant role of endocannabinoid system in development and pathophysiology of human melanoma and imply the role of cannabinoids in the treatment of this cancer. Particular substances exert different actions on endocannabinoid system, resulting in various effects on melanoma. Some of them present off-target mechanisms or involve less studied receptors. They can be divided into natural substances, with broader spectrum of action and synthetic ligands with often more accurate action.

## Discussion

The knowledge about the role of cannabinoids in health and disease is still progressing. Due to encouraging results of application of the cannabinoids in various cancers and their relatively low toxicity, they began to gain interest in the field of melanoma treatment.

There are couple of factors that impact the signaling of cannabinoids. They are exerting their actions through various signaling pathways, not only by widely described CB receptors. These actions may be mediated via adenosine mechanisms, strychnine-sensitive glycine receptors, GPR55, GPR119 and 5-HT (serotonin) receptors or TRP channels [23, 46]. Moreover, cannabinoids are likely to interact with various signaling pathways [42]. The full action of cannabinoids may be a result of simultaneous activation of various receptors present on the same cell [71]. Cannabinoids also exert different effects on various tissues and even cell lines and some effects vary between in vitro and in vivo models [57, 62, 64, 65, 70]. For example: cells of A375 melanoma line express high levels of COX-2, which may affect the actions of particular cannabinoids like AEA or AM251 [27, 69], and AEA produces effects in in vitro model, but not in vivo of HT168-M1 cell line [57]. Various levels of expression of receptors and pathways in particular tissues are likely not to be the only explanation of this phenomenon. The role of tumor microenvironment cannot be excluded [62, 64]. In contrast, in HCmel12 cell line, THC produced effects in in vivo models, but not in in vitro. Potential explanation was a low expression level of CB1 and CB2 receptors in this cell line, interaction with immune cells and tumor microenvironment [62]. Some cannabinoids can inhibit tumor growth in vivo by antagonizing the infiltration of immune cells in characteristic pro-inflammatory microenvironment. THC modulates the activity and number of macrophages, natural killer cells and decreases function of T-cells [62]. These all taken together suggest that molecular mechanisms of cannabinoids’ anti-tumor actions are complex and not fully understood.

In the case of melanoma, most important actions of cannabinoids described so far are decrease of cells viability by increase of apoptosis, necrosis [70] and cell cycle arrest [57, 69].

**Fig. 1:**
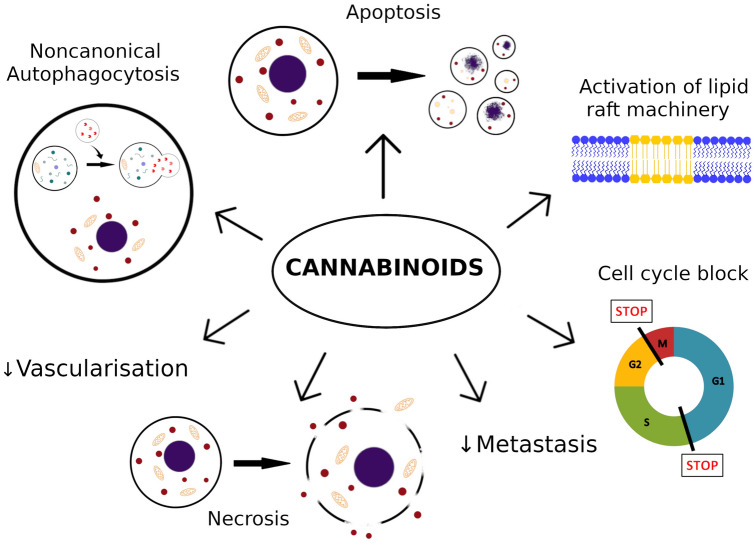
Summary of destructive mechanisms of actions exerted by cannabinoids on melanoma

Potential anti-proliferative mechanisms involved in melanoma cell apoptosis caused by cannabinoids are caspase-dependent apoptotic pathway [26, 27, 62, 65], inhibition of AKT and dephosphorylation of retinoblastoma protein [64], ERK phosphorylation [26], lipid raft machinery [26, 27] and activation of autophagy [65] (Fig. 1).

Moreover, cannabinoids slow down disease progress by reduction of metastasis and tumor vascularization [4, 63, 64, 66]. Potential mechanisms involve inhibiting epidermal growth factor receptors (EGFR) and vascular endothelial growth factor receptors (VEGFR) in tumors, induction of changes in endothelial cells and down-regulation of molecules involved in adhesion like: intercellular adhesion molecule (ICAM), vascular cell adhesion molecule (VCAM) and metalloproteinases (MMPs) [4, 63, 72].

The role of autophagy induced by cannabinoids remains not completely clear. It can either cause cell death, by autonomous death pathway, or activation of apoptosis. However, it can also act as a cytoprotective factor or source of energy to resist cell death caused by cytotoxic therapy. It seems to vary with cannabinoids and the cell types [65, 73]. In melanoma, cannabinoids seem induce non-canonical autophagy that leads to cell death via apoptosis. This mechanism involves TRIB3 and leads to inhibition of Akt/mTORC1 signaling [65]. These diverse mechanisms of action of cannabinoids may serve as a potential therapy in case of triple negative melanomas (harboring wild-type BRAF, NRAS and PTEN), which do not respond to a novel immunotherapy.

Due to large variety of cannabinoids, there are many potential derivatives, which may be found useful in the therapy of melanoma. Recently, some new cannabinoid derivatives and CB receptors ligands have emerged, that may have therapeutic potential in various cancers [28]. Some of them are reported to be devoid of any major side effects. For instance, a recently described orally active cannabinoid peptide (Pep19—the ligand of CB1), lacks of effects on central nervous system or heart rate alterations [74]. There is a high need of further both, in vitro and in vivo studies to determine their interactions with widely used drugs.

Other potentially useful compounds involved in endocannabinoid system function may be inhibitors of enzymes degrading cannabinoids. For example, the enzymatic hydrolysis of endocannabinoids like AEA and PEA is done mainly by the fatty acid amide hydrolase (FAAH) and the *N*-acylethanolamine-hydrolyzing acid amidase (NAAA) [75, 76]. Positive effects of inhibiting these enzymes may be observed also in case of melanoma treatment [70]. Moreover, FAAH inhibitors have been described to spare adverse psychotropic effects [77, 78].

Cannabinoids may complement currently used melanoma pharmacotherapies and counteract several side effect of chemiotherapeutics. Besides potential anti-tumor actions of cannabinoids, the compounds also produce different effects, which can potentially improve quality of life in patients with cancers. Most of this data, however, is limited and often requires studies and trials of higher quality. For instance, the cannabinoids are potentially efficient drugs in decreasing cancer pain and, therefore, increasing the patients’ quality of life [79, 80]. Cannabinoids have been also implied in treatment of cachexia, where they increased the patients’ appetite [81]. Application of cannabinoids may be beneficial in some patients with sleep disorders [82]. Some cannabinoids have been found useful in treatment of vomiting associated with chemotherapy, especially in patients, who did not respond to traditional anti-emetic drugs [83]. It is important to keep in mind that some people, often adolescents and young adults, may use cannabinoids from their own initiative as recreational drugs [84].

Despite potential mitigating action of cannabinoids on side effects of other melanoma pharmacotherapies, data about complementing these compounds together remain limited. So far one study assessed a problem of such interactions and involved nivolumab, the programmed death-1 (PD-1) inhibitor, one of main anti-melanoma agents. The usage of cannabis containing different concentrations of THC and CBD during immunotherapeutic treatment with nivolumab decreases response rate, without affecting progression-free survival and overall survival. There was no significant relation to cannabis composition, dose or the way of use [85]. However, the main limitation was a retrospective model of the study with relatively small group of patients and a non-representative sample of patients with melanoma. Results have suggested that combining these drugs should be carried out with caution and further studies are required to assess the way of combining cannabinoids with currently used anti-melanoma immunotherapy.

The main limitation of this comparative overview is small number of the available studies, different types of used melanoma cell lines, various incubation times and techniques used. In in vivo studies, each murine model of melanoma has its advantages and disadvantages, which may impact the results [86]. For example, xenograft models lack immune system, therefore, they do not provide the proper microenvironment for the tumor [87]. All these factors seem to be important due to great variety of genetic diversity of melanoma.

## Conclusion

Cannabinoids seem to be promising agents in the setting of melanoma treatment. However, due to limited number of studies and data available their role in modulation of this tumor progression remains unclear. They are likely to be part of multi-targeted drugs combination therapy, rather than the single drug treatment. More advanced molecular studies are necessary to evaluate the exact role of cannabinoids in melanoma treatment. Only few of the conducted studies determined interactions of cannabinoids with currently used melanoma therapies, therefore, it would be beneficial to investigate this problem. It requires further investigation through both, in vitro and in vivo studies, to determine the exact role of cannabinoids in melanoma treatment. Undoubtedly, the treatment of melanoma requires multi-targeted drugs combination strategy, potentially including cannabinoids.
